# Different facets of tree sapling diversity influence browsing intensity by deer dependent on spatial scale

**DOI:** 10.1002/ece3.3217

**Published:** 2017-07-26

**Authors:** Bettina Ohse, Carolin Seele, Frédéric Holzwarth, Christian Wirth

**Affiliations:** ^1^ Institute of Systematic Botany and Functional Biodiversity Leipzig University Leipzig Germany; ^2^ German Centre for Integrative Biodiversity Research (iDiv) Halle‐Jena‐Leipzig Germany

**Keywords:** biodiversity, foraging theory, forest inventory data, forest regeneration, plant–herbivore interactions, species composition, species identity, species richness, temperate forest, ungulate browsing

## Abstract

Browsing of tree saplings by deer hampers forest regeneration in mixed forests across Europe and North America. It is well known that tree species are differentially affected by deer browsing, but little is known about how different facets of diversity, such as species richness, identity, and composition, affect browsing intensity at different spatial scales. Using forest inventory data from the Hainich National Park, a mixed deciduous forest in central Germany, we applied a hierarchical approach to model the browsing probability of patches (regional scale) as well as the species‐specific proportion of saplings browsed within patches (patch scale). We found that, at the regional scale, the probability that a patch was browsed increased with certain species composition, namely with low abundance of European beech (*Fagus sylvatica* L.) and high abundance of European ash (*Fraxinus excelsior* L.), whereas at the patch scale, the proportion of saplings browsed per species was mainly determined by the species’ identity, providing a “preference ranking” of the 11 tree species under study. Interestingly, at the regional scale, species‐rich patches were more likely to be browsed; however, at the patch scale, species‐rich patches showed a lower proportion of saplings per species browsed. Presumably, diverse patches attract deer, but satisfy nutritional needs faster, such that fewer saplings need to be browsed. Some forest stand parameters, such as more open canopies, increased the browsing intensity at either scale. By showing the effects that various facets of diversity, as well as environmental parameters, exerted on browsing intensity at the regional as well as patch scale, our study advances the understanding of mammalian herbivore–plant interactions across scales. Our results also indicate which regeneration patches and species are (least) prone to browsing and show the importance of different facets of diversity for the prediction and management of browsing intensity and regeneration dynamics.

## 1 INTRODUCTION

Deer browsing on tree saplings severely hampers regeneration in temperate forests of Europe and North America (Ammer, Vor, Knoke, & Wagner, 2010; Kittredge & Ashton, 1995; Putman, 1996; Rooney & Waller, 2003). By negatively impacting the saplings’ vitality (Kupferschmid & Bugmann, 2008), browsing is a major impediment for successful regeneration (Ammer & Vor, 2013; Kuiters & Slim, 2002). To better understand mechanisms of browsing intensity, we can refer to foraging theories, which state that browsing intensity should be largely determined by the quantity and quality of the forage (Shipley & Spalinger, 1995; Stephens & Krebs, 1986). However, quality effects on herbivory may operate via different mechanisms of species diversity, such as species richness, species composition, and species identity, as has been shown for insect herbivory (Haase et al., 2015; Jactel, Brockerhoff, & Duelli, 2005; Schuldt et al., 2010; Sobek, Scherber, Steffan‐Dewenter, & Tscharntke, 2009). Also, browsing is a decision at different spatial scales (Mysterud, Lian, & Hjermann, 1999; Rautio, Kesti, Alm Bergvall, Tuomi, & Leimar, 2008; Senft et al., 1987). Food selection patterns of deer have been described as a hierarchical process, as selectivity can occur both between patches and within patches (Alm Bergvall, Rautio, Kesti, Tuomi, & Leimar, 2006; Alm Bergvall, Rautio, Sirén, Tuomi, & Leimar, 2008). Both aspects, the influence of different facets of diversity on browsing and how they operate at different scales of foraging selectivity are still largely unknown.

At a regional forest scale, a deer searching for food needs to decide which patch to select for browsing. It decides by optimizing quantity and quality of the food intake (Illius, Duncan, Richard, & Mesochina, 2002). A high quantity of forage is obtained in patches with a high density of vegetation (Shipley & Spalinger, 1995). In particular, roe deer, having a small rumen capacity (Hofmann, 1989), are restricted in their ability to digest large volumes of material (Westoby, 1978). Thus, they need to carefully balance food quantity and quality. Qualitative differences between patches can be described by species composition and are mainly related to the relative abundance of high‐quality versus low‐quality species (Augustine & McNaughton, 1998). It has been shown, for instance, that red deer select vegetation patches with high relative abundances of palatable species (Bee et al., 2009). Deer might also encounter patches of different species richness, that is, diet diversity. A diet based on a single species can rarely satisfy all nutritional needs. Rather, a diverse diet should be advantageous to the animal, because plant species differ in their nutritional content. Also, most tree species contain certain secondary metabolites and feeding on a diverse diet avoids over‐ingestion of a particular secondary metabolite (Freeland & Janzen, 1974; Provenza, Villalba, Dziba, Atwood, & Banner, 2003). In a boreal forest experiment, high species richness increased moose browsing (Milligan & Koricheva, 2013), yet it is to be examined, whether sapling species richness likewise increases browsing probability by deer in temperate forests. Also, the relative importance of species richness and species composition has not been examined in the context of mammalian herbivory.

At a patch scale, that is, once the deer has decided for a certain patch to forage in, it needs to decide which individual plant to select for browsing. As plant species differ in their overall palatability, browsing mammals likely prefer high‐quality over low‐quality plant species. Such species identity effects on forage selection of deer have been shown in a variety of studies (Boulanger et al., 2009; Kuijper et al., 2010; Maillard & Picard, 1987; Modrý, Hubený, & Rejsek, 2004). However, preference rankings of tree species differ and it has been shown that preference by deer of single tree species is often conditional on the neighboring tree species. For example, in mixed coniferous/deciduous forests, deer often prefer beech over spruce or pine, but avoid beech if it occurs in mixture with other deciduous trees, such as sycamore maple or ash saplings (e.g., Ammer et al., 2010). Hence, preference rankings between species could also be changed by the species composition of the community matrix of the patch, also called associational effect (Alm Bergvall et al., 2006). Moreover, with increasing species richness, patches might experience lower herbivory rates because balanced nutrition may be reached faster during the foraging process than in species‐poor patches. To our knowledge, these facets of diversity, namely species identity, species composition, and species richness, have not been related systematically to browsing decisions at the patch scale, yet. Additionally, the mere fact of a tree species being rare, that is, of low relative abundance, has been suggested by many foresters to lead to a higher browsing intensity of those species. On the one hand, rare species might provide rare nutrients, making them more attractive to deer; on the other hand, they are also more difficult to detect for deer. Observational studies in temperate forests revealed that browsing was more intense on more relatively rare species (Čermák, Horsák, Špiřík, & Mrkva, 2009; Seele, 2011), whereas in New Zealand forests, browsing by red deer was not related to the rarity of the focal species (Bee et al., 2009). However, the interplay between species rarity and identity is still unclear.

By extending foraging theories, we examine in this study the effects of different facets of diversity on browsing intensity by deer across different scales of foraging selectivity. We assessed the importance of species richness, species identity, and species composition relative to effects of forage quantity on browsing intensity at two spatial scales: regionally between patches and locally within patches. We used forest and regeneration inventory data from the Hainich National Park, a mixed deciduous forest in central Germany. When working with observational data, other factors influencing deer browsing need to be taken into account, such as forest site characteristics, including the light regime (Gill & Beardall, 2001), and potential disturbance of the feeding animals by, for example, hiking trails (Möst, Hothorn, Müller, & Heurich, 2015). Other studies also included topographic information (elevation, slope, aspect) (Campbell, Laseter, Ford, Odom, & Miller, 2006; Vospernik & Reimoser, 2008). Hence, in our study, we control for these forest stand and environmental parameters. We approximated species composition and thus forage quality of each patch using the relative abundances of the three most common tree species, which differ in their forage quality based on measured traits relevant for browsing. By developing a model for each of the two scales, we tested the following hypotheses:

1. At the regional scale
  - The number of saplings (forage quantity) is the most important driver of patch selection probability, followed by species composition (forage quality), and species richness.
  - Patch selection probability is high for patches with a species composition containing a high relative abundance of high‐quality species, as well as for patches with a high species richness (mixed diet).
2. At the patch scale
  - Species identity and species composition (i.e., the quality of individual saplings and of the surrounding matrix of neighboring saplings) are the main drivers of selection of individual species.
  - However, the identity of the species will be more important than species composition, forage quantity, or species richness.
  - Moreover, we assume rare species in a patch to be browsed proportionally more than abundant species, irrespective of their identity.

## 2 MATERIALS AND METHODS

### 2.1 Study area

This study was carried out in the Hainich National Park in central Germany. The National Park is named after the prevailing low mountain range “Hainich” (mean elevation 400 m a.s.l.), which is built of Triassic limestone covered with Pleistocene loess (Grüneberg, Schöning, Kalko, & Weisser, 2010). The area has a mean annual temperature of 7°C and a mean annual precipitation of 650 mm (Fischer et al., 2010). The Hainich is the largest contiguous deciduous forest area in Germany (13,000 ha). The Hainich National Park comprises 7,500 ha and is mainly covered by temperate deciduous forest (67% of the area), which is dominated by European beech (*Fagus sylvatica* L.), with several admixed deciduous tree species, such as European ash (*Fraxinus excelsior* L.), sycamore maple (*Acer pseudoplatanus* L.), Norway maple (*A. platanoides* L.), European hornbeam (*Carpinus betulus* L.); and, with slightly lower abundances, lime (*Tilia* spec.), wych elm (*Ulmus glabra* Huds.), and oak (*Quercus petraea* agg., *Q. robur* L.). Large parts of the National Park were managed as coppice with standards in the 18th century and were partly converted to selection forests in the 19th century (Wäldchen, Schulze, Mund, & Winkler, 2011). Some areas were used as military training ground for several years during the 20th century, but most areas have not been managed since the 1950s (A. Henkel, Hainich National Park, personal communication). As at 2016, about 5,000 ha is unmanaged forest. There are three wild ungulate species in the area, whose individual numbers were estimated by spotlight counts as follows (Heinze, 2010): roe deer (*Capreolus capreolus* L.) with 12.4 individuals per 100 ha, red deer (*Cervus elaphus* L.) with 3.1 individuals per 100 ha, and fallow deer *(Dama dama* L.) with 2.3 individuals per 100 ha. Spotlight counts provide good estimates for red deer numbers, but tend to underestimate roe and fallow deer densities by about 30% compared to pellet counts or footprint counts in snow (Heinze et al., 2011). Following this correction, roe deer would be the most abundant deer species in the area (with 16.1 individuals, compared to 3.1 red deer and 3.0 fallow deer per 100 ha). Although roe deer has a smaller body size and thus consumes less biomass per individual than red deer and fallow deer, it is the only pure browser, whereas red deer and fallow deer are mixed or intermediate feeders (Clauss, Lechner‐Doll, & Streich, 2003; Hofmann, 1989). However, as data comparing the absolute biomass intake from tree saplings by the three deer species is lacking, we cannot precisely attribute browsing of tree regeneration to either of the deer species.

### 2.2 Inventory data collection

A raster‐based forest inventory was conducted by the National Park across the entire area from November 2009 to May 2011, with a plot grid space of 200 m. For each plot, the natural regeneration was inventoried on the NE quarter of a circle of 3 m radius (i.e., ca. 7 m²). Browsing was recorded on all saplings in the plot in three height classes. However, we decided to only use the data on saplings in the first two height classes (20–50 and 50–130 cm), as the third sapling height class comprises 130 cm height to 7 cm dbh (several meters high) and is thus for the most part out of the reach of deer. For all saplings, the number of browsed and unbrowsed saplings per species was recorded, while “browsed” meant the terminal bud was browsed in the year of inventory. Terminal bud browsing is a regularly used measure in forest inventories, as terminal buds are in most cases the first part of a sapling to be browsed, while browsing on lateral buds only is very unlikely. Therefore, we consider terminal bud browsing a sufficient measure to assess whether a tree has been selected by deer or not. Due to difficulties in specifying saplings of certain species in the field and thus the occurrence of genus level records for some, these genera and their species were pooled to genus level: *Quercus*,* Tilia*, and *Ulmus*. We calculated the total number of saplings per plot (sapling density), the relative abundance per species (or genus, respectively), as well as species richness and Shannon diversity, per plot. The Shannon diversity index takes into account the species number as well as their abundances. We also calculated the frequency of species across all plots and only considered species with an *across‐plot* frequency of 20 and higher for further analyses. There is no distinct shrub layer in the area, so we refrained from using shrub species in our analysis. The final dataset resulted in 11 tree species and a total of more than 9,500 individual saplings being included in the modeling procedure.

During the same inventory, stand‐level data were recorded on a 100 m × 100 m plot around the plot center, and comprised elevation, slope, and aspect. Crown closure was recorded in four categories (from densely packed crowns to large gaps between crowns). Disturbance to deer by human activities was quantified using a National Park wide map of hiking trails and calculating the distance of each plot center to the nearest hiking trail. The final dataset used for analyses comprised 817 plots. Variables and their summary statistics are shown in Table 1.

**Table 1:** Summary statistics for the inventory data used in this study (817 plots)

Variable	Min	Median	Max	Variable	Frequency across plots
Number of saplings per plot	1	6	134	Species identity	
Species richness	1	2	8	*Acer campestre*	46
Rel. abundance beech	0	0.29	1	*Acer platanoides*	170
Rel. abundance ash	0	0.01	1	*Acer pseudoplatanus*	370
Rel. abundance sycamore	0	0	1	*Carpinus betulus*	100
Elevation [m]	247	381	499	*Fagus sylvatica*	528
Slope [°]	0	5	51	*Fraxinus excelsior*	424
Aspect [gon]^a^	0	120	395	*Populus tremula*	21
Distance to trail [m]	0.04	272	2,327	*Prunus avium*	36
Crown closure	**Frequency across plots**			*Quercus* spec	28
Sparse		61		*Tilia* spec	37
Loose		134		*Ulmus* spec	23
Closed		529			
Crowded		92			

a Transformed to linear values of northness and eastness for subsequent statistical analyses.

### 2.3 Statistical analyses

As browsing selectivity was assumed to occur at two spatial scales, between patches and within patches, we used a hierarchical modeling approach. In a first, regional‐scale model, all plots were modeled as either being browsed (any browsing sign on any species, 371 plots) or not (no single browsed individual, 446 plots), using a generalized linear model (GLM) with a binomial error distribution. In a second, patch‐scale model, only the browsed plots were analyzed, and the proportion of individuals browsed per species was modeled as a dependent variable, using a GLM with a beta‐binomial error distribution. This type of error model has been used previously for modeling forest health parameters, where proportions close to zero or one are more likely (Zarnoch, Anderson, & Sheffield, 1995). The beta‐binomial GLM accounts for this type of over‐dispersion and is implemented in the R package gamlss (Stasinopoulos & Rigby, 2007).

In both the regional‐scale as well as the patch‐scale model, we included as predictors the total number of saplings per plot to describe forage quantity, and the sapling species richness to describe the diversity of the diet. Although Shannon index was slightly less correlated with sapling numbers than species richness (see Table S1), we refrained from using Shannon index, because it contains species abundances, which we intended to analyze separately. There are two common ways to capture species composition to use as predictor in a model: (1) include each realized combination of species, which is only possible in an experimental setup with predefined species compositions and equal abundances of species within a composition (e.g., Milligan & Koricheva, 2013); or (2) include each species with a binary coding as a single predictor, which is only possible with a limited amount of species (e.g., Hantsch, Braun, Scherer‐Lorenzen, & Bruelheide, 2013). Both approaches are not feasible in an observational study such as this one with more than 10 species in any combinations and with varying abundances. Also, approaches meant to reduce the dimensions of many variables, such as principal coordinate analysis (PCoA), were not sufficient to adequately capture species composition in this study (see Appendix S1). As a proxy of the effect of species composition, we therefore used the relative abundances of the three main species in the regeneration layer *F. sylvatica*,* F. excelsior*, and *A. pseudoplatanus*. As these species are known to differ markedly in several browsing relevant traits (see Appendix S2), their relative abundances approximate food quality of a plot as a whole (regional scale) and represent associational effects of the community matrix within a plot (patch scale). For quantifying associational effects, it would be even more desirable to calculate palatability of target species *relative* to the palatability of the neighboring species (as done by, e.g., Bee et al., 2009); however, we found it impossible to derive an independent measure of neighbor palatability without introducing circularity. Slope, aspect, and elevation were used as abiotic environmental variables. Aspect was transformed into continuous measures of northness and eastness, taking the cosine and sine, respectively, from the radiant. The stand‐level crown closure was included as a proxy for light availability to the saplings, and the distance to the nearest hiking trail was used as a measure for potential disturbance of deer by human activities. All continuous variables were scaled between zero and one.

At the regional scale, that is, for modeling the probability that a plot has been browsed, we additionally included first‐order interaction terms between the number of saplings per plot and species composition (each of the relative abundances of the three main species) to account for interaction effects of quantity and quality of the diet. At the patch scale, that is, for modeling the proportion of saplings per species that have been browsed in a plot, we included “species” as a categorical variable to address species identity effects, that is, to be able to quantify species‐specific browsing probabilities. We also included first‐order interaction terms to allow certain effects to vary per species, that is, we included interactions between the target species identity and its own relative abundance, to examine whether a “rarity effect” exists only for certain species or in general. We also included interactions between species identity and species richness, in order to test whether the identity effect varies across the richness gradient. Both models with their respective predictor variables are shown in Table 2.

**Table 2:** Model approach addressing different levels of browsing selectivity. The regional‐scale model addresses selectivity between plots, the patch‐scale model addresses selectivity within plots. Predictor variables are shown for both models

Regional‐scale model	Patch‐scale model
*Response*	*Response*
Probability that a plot is browsed (yes/no; binomial GLM)	Proportion of individuals browsed (0–1; beta‐binomial GLM)
*Predictors*	*Predictors*
	Target species identity
	Target species relative abundance
Species richness	Species richness
Species composition	Species composition
Relative abundance beech	Relative abundance beech
Relative abundance ash	Relative abundance ash
Relative abundance sycamore	Relative abundance sycamore
Number of saplings per plot	Number of saplings per plot
Environment	Environment
Crown closure	Crown closure
Elevation	Elevation
Aspect (northness, eastness)	Aspect (northness, eastness)
Slope	Slope
Distance to next hiking trail	Distance to next hiking trail
Quantity‐quality interaction effects	Species‐specific interaction effects
Number of saplings per plot × rel. abundance beech	Target species identity × Species richness
Number of saplings per plot × rel. abundance ash	Target species identity × Target species relative abundance
Number of saplings per plot × rel. abundance sycamore	

Variable selection for the regional‐scale model was performed by backward selection, dropping nonsignificant variables based on chi‐square tests. To test for spatial autocorrelation, a Moran's I test was performed on the residuals (Cliff & Ord, 1972; Moran, 1950) using the R package “spdep” (Bivand, 2010). The model prediction accuracy was assessed by AUC, which is the area under the receiver operating curve (ROC, Bradley 1997). The ROC relates the true‐positive rate (number of correctly predicted browsed plots divided by total number of plots) to the false‐positive rate (number of falsely predicted browsed plots divided by total number of plots). Variable selection for the patch‐scale model was performed by backward selection, dropping nonsignificant variables based on chi‐square tests. Model fit was assessed by plotting residuals against fitted values as well as against the explanatory variables. For each of the two final models, the importance of the single predictors was ranked according to the change in AIC upon deletion of each explanatory variable from the minimal adequate model.

## 3 RESULTS

### 3.1 Regional‐scale model

**Figure 1:**
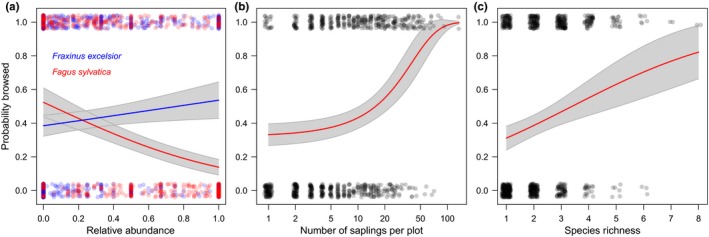
Regional‐scale browsing probability of a plot, depending on (a) species composition (relative abundance of beech (*Fagus sylvatica* L.)—red, and ash (*Fraxinus excelsior* L.)—blue), (b) forage quantity, that is, number of saplings per plot (note logarithmic *x*‐axis), and (c) species richness. Dots (jittered) show the original data; lines show predictions with 95% confidence intervals (keeping all other variables constant at their medians)

The number of saplings per plot, that is, forage quantity, was not the most important predictor for the probability that a plot was browsed. Instead, species composition, especially the relative abundance of beech, was most important. As expected, species richness was of minor importance (Table 3). A low share of beech, high sapling densities, and a high species richness of the regenerating saplings increased the probability that a plot was browsed (Figure 1a–c). More specifically, a 20% increase in the share of beech led to ca. 10% decrease in the browsing probability of a plot. If plots contained less than 20 saplings, their browsing probability was below 0.5, beyond 20 saplings plots got more attractive and browsing probability increased up to 1 for plots with more than 50 saplings. Mono‐specific plots had a browsing probability of ca. 0.3, while plots with the highest species richness (eight species) had a browsing probability of 0.8. Moreover, elevation, crown closure, and distance to a trail influenced the browsing probability (Table 3). Plots at relatively high elevations, under open crowns, and close to hiking trails were more likely to be browsed (see Appendix S3). There were significant positive interaction effects between sapling number and the relative abundances of both ash and sycamore maple, meaning that the “quality effect” of the two species becomes slightly more pronounced with higher sapling quantities. However, both interaction effects were ranking last in the variable importance. The same model without the interaction terms was only slightly worse (residual deviance 861.48 vs. 871.43, *p* = .02, Chi‐square test); hence, all other results shown here are based on the more parsimonious model without the two interaction terms. The variable importance ranking of the final model was not sensitive to replacing species composition (relative abundances of beech and ash) with a different proxy of forage quality (community‐weighted mean palatability) of each plot (see Appendix S4).

**Table 3:** Relative importance of predictors and summary of the coefficients for the final regional‐scale model predicting the browsing probability per plot. Relative importance of predictors was quantified by delta AIC (change in AIC upon single term deletion, compared to the final model with AIC** = **891.4). Effect sizes are standardized (continuous variables were scaled between 0 and 1). Diversity facets are in bold

Variable	delta AIC	Estimate	*SE*	*p*‐Value
(Intercept)		−2.25	0.40	<.001
**Species composition** (relative abundance of beech)	50.0	−1.92	0.28	<.001
Elevation	35.6	2.47	0.42	<.001
Number of saplings per plot	28.8	6.30	1.25	<.001
Crown closure	12.9			
Closed		0.63	0.28	.022
Loose		1.32	0.33	<.001
Sparse		1.08	0.40	.007
**Species richness**	11.5	2.32	0.64	<.001
Distance to trail	5.2	−1.45	0.55	.008
**Species composition** (relative abundance of ash)	3.3	0.61	0.27	.022

With the final regional‐scale model, we were able to correctly classify 72.8% of the plots. More specifically, 257 of 371 plots were correctly classified as being browsed, and 337 of 446 plots were correctly classified as not being browsed. As 114 plots were falsely predicted as browsed, this resulted in an AUC value of 0.80 (AUC ≥ 0.8 is considered excellent, Hosmer & Lemeshow, 2000). Spatial autocorrelation as tested using Moran's I was low across the entire study area (global Moran's *I* = 0.04, *p*‐value = .003).

### 3.2 Patch‐scale model

**Figure 2:**
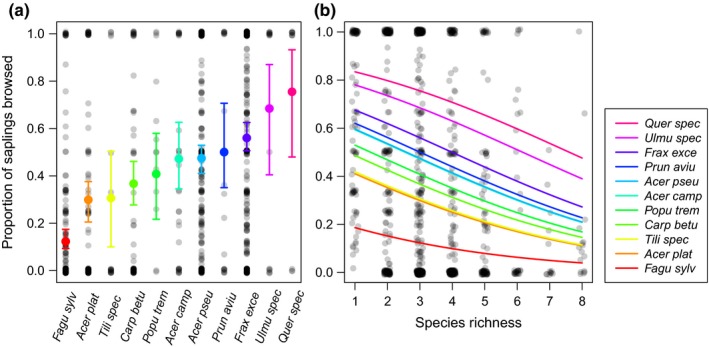
Patch‐scale proportion of saplings browsed per species, depending on (a) species identity, and (b) species richness. Species are ordered from left to right (a), or from bottom to top (b), respectively, according to increased browsing proportion (for fulll species names see Table 4). Gray dots (jittered) show the original data; colored dots and lines show predictions (keeping all other variables constant at their medians)

We hypothesized that species identity and species composition (of the matrix) would have the strongest influence on the proportion of individual saplings per species browsed per plot. Indeed, species identity was by far the most important predictor for the proportion of saplings browsed per species, but unexpectedly, species composition (i.e., relative abundances of the three main species beech, ash, and sycamore maple) was not significant in the model (Table 4). The proportion of saplings browsed per species was highest for oak species (ca. 80%) and lowest for beech (ca. 10%). Figure 2a shows all 11 species with their specific proportion of saplings browsed, displaying a “preference ranking” from low to high browsing proportions. Surprisingly, species richness was the second most important predictor at the patch scale and the proportion of saplings browsed per species was generally lower when more species were present in the plot (Figure 2b). For instance, the proportion of saplings browsed of *P. avium*,* A. pseudoplatanus*,* A. campestre*,* P. tremula* (medium browsed species), decreased from ca. 60% in monospecific plots to ca. 30% in 8‐species plots. Elevation, crown closure, distance to trail, and eastness were of minor importance for predicting browsing proportions of saplings at the patch scale (Table 4). Contrary to our hypothesis, rarity (relative abundance) of a target species did not significantly influence its proportion of saplings browsed in a plot. There was no significant influence of the number of saplings in a plot, nor of slope, on the proportion of saplings browsed.

**Table 4:** Relative importance of predictors and summary of the coefficients for the final patch‐scale model predicting the proportion of saplings browsed per species. Relative importance of predictors was quantified by delta AIC (change in AIC upon single term deletion, compared to the final model with AIC = 2537.9). Effect sizes are standardized (continuous variables were scaled between 0 and 1). Diversity facets are in bold

Variable	delta AIC	Estimate	*SE*	*p*‐Value
(Intercept)		−2.48	0.32	<.001
**Species identity**	214.0			
* Acer campestre*		1.85	0.31	<.001
* Acer platanoides*		1.11	0.23	<.001
* Acer pseudoplatanus*		1.86	0.16	<.001
* Carpinus betulus*		1.42	0.26	<.001
* Fraxinus excelsior*		2.21	0.16	<.001
* Populus tremula*		1.59	0.42	<.001
* Prunus avium*		1.97	0.40	<.001
* Querus* spec.		3.09	0.77	<.001
* Tilia* spec.		1.15	0.51	.026
* Ulmus* spec.		2.74	0.51	<.001
**Species richness**	35.0	−1.71	0.27	<.001
* *Distance to trail	18.9	−1.11	0.24	<.001
* *Elevation	11.3	0.91	0.24	<.001
* *Crown closure	10.3			
Closed		0.46	0.22	.034
Loose		0.76	0.24	.001
Sparse		1.02	0.29	<.001
Eastness	4.3	0.47	0.19	.016

## 4 DISCUSSION

With this study, we aimed to determine the relative importance of different facets of sapling diversity on browsing intensity by deer in relation to common drivers of ungulate foraging, such as food quantity and environmental factors, at two scales of foraging. At the regional scale, mainly species composition and sapling quantity determined browsing probability, whereas at the patch scale, species identity was the most important factor determining the proportion of browsed saplings. Species richness was less important at the regional scale, but of relatively high importance at the patch scale, and had contrasting effects at the two scales. Our results support existing knowledge on the importance of forage quantity and quality to browsing animals, but expand these theories by showing that facets of diversity can be equally or even more important than forage quality and quantity, depending on the spatial scale considered.

### 4.1 Regional scale

At the regional scale, species composition was the most important predictor to determine browsing probability of a patch, which is in line with our hypothesis and with recent studies highlighting the importance of species composition for foraging decisions by browsing mammals, such as moose (Milligan & Koricheva, 2013) or red deer (Bee et al., 2009). Koricheva, Mulder, Schmid, Joshi, and Huss‐Danell (2000) showed that the effect of composition is due to the presence of a specific plant or plant functional type. We found that deer selectively browsed patches with a low share of beech, corresponding to findings by Čermák et al. (2009), and/or a high share of ash, although the beech effect was much more pronounced. Beech has been reported as a species of comparatively poor nutritional quality to deer, mainly because of its high fiber and silica content and low protein content (González‐Hernández & Silva‐Pando, 1999), which likely deters deer from browsing beech dominated patches. High sapling numbers increased the browsing probability of a patch, thus supporting previous foraging theories (Spalinger & Hobbs, 1992; Stephens & Krebs, 1986), although forage quantity was a less important predictor than forage quality. There was no interaction effect between sapling numbers and the share of beech in a patch; hence, a trade‐off between high diet quantity but low quality (Illius et al., 2002) is unlikely in our study. Potentially, patches with a high number of saplings might also be selected by deer due to better protection, leading to less vigilance and hence more time for feeding (Benhaiem et al., 2008). However, as there are no predators in the area and hunting is very restrictive, we assume protection by high sapling numbers to be less important than the mere forage quantity affect.

The low importance of species richness might be due to its relatively high correlation with sapling numbers (Table S1), such that the effect of increased browsing probability of a patch due to more saplings mitigates the adjunctive effect of more species. Rarefaction has been proposed to disentangle dependencies of species richness on individual numbers (Simberloff, 1972), but has also been criticized (Forbes, Schauwecker, & Weiher, 2001) and seems unsuitable in our case because of relatively low numbers of saplings per plot (minimum 1, median 6). A mere sampling effect, that is, a higher browsing probability of species‐rich patches due to an increased probability of occurrence of palatable species, is also unlikely. As the probability of occurrence of unpalatable species should increase likewise with species richness, it is rather a question of which species, palatable or unpalatable, gains dominance (Hector, Bazeley‐White, Loreau, Otway, & Schmid, 2002). In this study, dominance, that is, sapling abundance per species, and predicted browsing proportion per species (as a proxy for palatability) did not correlate across the National Park (Spearman correlation = 0.46, *p* = .15). Hence, the slightly higher browsing probability of species‐rich patches is not due to a mathematical sampling effect, but can be attributed to species richness per se. We propose that the higher browsing probability of species‐rich patches is likely due to a more diverse diet being beneficial to deer (Augustine & McNaughton, 1998). However, when considering net‐effects of species richness on browsing intensity across both scales, the patch‐scale effect dominates and leads to a net decrease in browsing proportions with increasing species richness (Appendix S5).

The environmental variables elevation and crown closure were of similar importance as forage characteristics. Elevation was an important predictor of browsing in previous studies (Campbell et al., 2006; Vospernik & Reimoser, 2008), and might in this study be a proxy for hidden variables that were not measured, such as forest stand density and cover, remoteness, or forest continuity. Low crown closure, that is, a more open stand, leaves more light to the tree saplings and can increase nutrient availability (Ritter & Vesterdal, 2006). We assume that this improves the saplings’ nutritional status, which makes saplings more attractive to deer and increases browsing probabilities.

### 4.2 Patch scale

At the patch scale, species identity had the strongest effect on the proportion of individual saplings browsed per species, whereas species richness was less important and species composition was not important at all. Roe deer can distinguish between alimentary items (Le Corre et al. 2008) and seem to decide precisely which species satisfy their nutritional needs best, resulting in a “preference ranking” of tree species. The preference ranking revealed in this study is similar to earlier rankings, with *Fagus sylvatica* and *Tilia* spec. often being least preferred (Čermák & Mrkva, 2003; Modrý et al., 2004; Didion, Kupferschmid, Wolf, & Bugmann, 2011; but see Boulanger et al., 2009) and *Quercus* spec and *Ulmus* spec most preferred (Boulanger et al., 2009; Didion et al., 2011; Maillard & Picard, 1987). In line with other studies, *F. excelsior* is slightly more preferred than *Acer* species, both ranking medium (Čermák & Mrkva, 2003; Maillard & Picard, 1987), while *Carpinus betulus* is found on either end of the preference rankings across studies (Boulanger et al., 2009; Kuijper et al., 2010) and ranks medium in our study. Differences in preference rankings can easily occur across forest sites within a region (Boulanger et al., 2009) or between seasons in the same site (Maillard & Picard, 1987), which makes generalizations difficult.

Although each species has its intrinsic palatability, associational effects of more or less palatable neighbors in the community matrix can modify a species relative palatability (Alm Bergvall et al., 2006; Hjältén, Danell, & Lundberg, 1993) and can thus change the browsing probability and the ranking of the individual target species. We hypothesized that species composition, measured as relative abundances of the dominant species beech, ash, and sycamore maple, and used as a proxy for palatability of the neighbor plants, would affect browsing proportions of individual target species. Although a high relative abundance of beech decreased the *total* proportion of all species’ saplings browsed per patch (see Appendix S6), we did not find such an associational effect on individual target species’ browsing proportions. Probably, there is no common effect of species composition on each of the target species, but we assume that species composition effects depend on each target species’ palatability relative to the neighboring plants’ palatability. Unfortunately, such a species‐specific effect of composition could not be tested explicitly, because of sparse replication (low frequency across plots) of some target species (see also Table 1). To test such neighbor‐quality effects on browsing intensity of single species, experimental approaches are necessary, for instance, planting phytometer saplings of certain target species within patches of different species composition. If palatability‐related traits of both the phytometer saplings and the surrounding community are known, one could quantify the neighbor‐quality effect on browsing intensity of single species.

Unexpectedly, species richness was the second most important predictor at the patch scale and browsing proportions of individual species were lower when growing within a species‐rich patch. Species richness can either increase herbivory (Milligan & Koricheva, 2013; Schuldt et al., 2010; Vehviläinen & Koricheva, 2006), or decrease herbivory (Vehviläinen & Koricheva, 2006, Sobek et al., 2009), depending on whether the herbivore is a generalist or specialist feeder, respectively (Castagneyrol, Giffard, Péré, & Jactel, 2013; Koricheva et al., 2006; Schuldt et al., 2010; Vehviläinen & Koricheva, 2006; Vehviläinen, Koricheva, & Ruohomäki, 2007). According to the resource concentration hypothesis (Root, 1973), it is more difficult for specialist (insect) herbivores to find host plants within more species‐rich patches, because of a dilution within nonhost plants. In contrast, in our study, species‐rich patches contain on average a higher proportion of palatable maple and a lower proportion of unpalatable beech (see Appendix S7). We suppose that reduced browsing proportions in species‐rich patches are due to the fact that patches with a higher tree species richness offer a more diverse and nutritionally satisfying diet and hence less food is needed (Westoby, 1974). In other words, fewer individuals are browsed and the risk of being browsed is spread across more species.

Contrary to our hypothesis, the rarity or relative abundance of a species within a patch did not significantly influence the species’ browsing proportion. Szmidt (1975) argued that roe deer rather feed on the most abundant tree species, that is, the ones they are most accustomed to. Several studies found an increased damage intensity with decreasing relative abundance, especially for palatable species (Čermák et al., 2009; Tixier et al., 1997). In our study, we did not find an interaction effect of species and their relative abundances, so we conclude that, independent of a species’ palatability, being rare is not per se a choice criterion, but that the species identity and its functional properties are the main choice criterion for deer.

### 4.3 Spatial scales

Brose and Hillebrand (2016) corroborated that biodiversity–ecosystem function relationships can change across spatial scales. The effects of diversity on ecosystem functions and processes are often examined at the scale of (experimental) plots, whereas, for example, foraging decisions and herbivory patterns scale with body size and thus the home range size of the animal. Although the avoidance or preference of species is consistent across the two scales of this study (here beech and ash, respectively), the contrasting effects of species richness at the two scales (increased browsing at regional scale, decreased browsing at patch scale) are likely a result of optimized hierarchical foraging decisions at different scales (Danell, Edenius, & Lundberg, 1991; Searle, Hobbs, & Shipley, 2005). Environmental conditions can further change selectivity patterns at even larger scales. In a review on browser—woody plant interactions, most studies from relatively nutrient poor systems (e.g., boreal forests) found low browsing selectivity between patches, but strong selection for individual palatable trees, while in resource‐rich environments (e.g., Białowieza Primeval forest) herbivores showed strong patch selection but were relatively unselective for individual plants within the patch (Cromsigt & Kuijper, 2011). Although the Hainich National Park is comparable in terms of tree species to the Białowieza Primeval forest, we found selectivity at both scales. In general, we advocate choosing the scale of future studies according to the scale at which certain biodiversity–ecosystem functioning relationships are ecologically relevant and to mirror hierarchical processes using hierarchical approaches.

## 5 CONCLUSIONS

Our study provides new insights into the effects of different facets of diversity on plant–herbivore interactions. We showed that the relative importance of species composition, species identity, and species richness on deer browsing varies with the spatial scale of foraging decision. The large effect of species identity at the patch scale implies that browsing is a selective process and can suppress long‐term regeneration of palatable species, favoring the least palatable ones (Augustine & McNaughton, 1998), in this case beech. By apparent competition, selective browsing can act as biotic filter that predetermines eventual canopy composition and related forest characteristics (Augustine & McNaughton, 1998). However, at a regional scale, we also found a strong compositional effect, which forest practitioners could take advantage of, for example, by mixing preferred tree species within avoided ones in the regeneration, as this seems to keep deer from choosing such patches for browsing. Reduced browsing of patches can, in turn, change interspecific competition between tree species, such that previously browsed species like maple gain a competitive advantage (Seele, 2011). At the patch scale, we showed that species richness reduces browsing, so that it would likewise be advisable to promote mixed rather than monospecific regeneration. Thus, according to our study, it may be possible, by supporting more mixed regeneration, to mitigate the selective, species identity‐dependent browsing pressure on the most preferred species. As rarity did not affect browsing intensity in this study, we argue that it is still worth promoting rare species despite browsing pressure, be it for conservational efforts or in forest management.

We acknowledge that drawing generalizations from case studies like this one, using data from one specific forest, is limited. We therefore advocate using the same approach with similarly structured forest inventory data, which should be widely available from other sites.

We furthermore suggest that forest models, which already have incorporated parameters related to species’ sensitivity to browsing (Didion et al., 2009), should also consider including different facets of sapling diversity. This would improve predictions of browsing effects on growth and survival of different species mixtures, be it to optimize future forest stands for economical use, climate change adaptation, or conservation.

Finally, we want to highlight that future studies addressing effects of biodiversity on plant–herbivore interactions as well as on other ecosystem processes should strive to incorporate all spatial scales relevant for the ecosystem process under study. Otherwise, opposing trends of diversity effects at different scales might not be detected adequately.

## AUTHOR'S CONTRIBUTIONS

BO, CS, and CW conceived the ideas and designed the methodology; BO and FH analyzed the data; BO led the writing of the manuscript. All authors contributed critically to the drafts and gave final approval for publication.

## DATA ACCESSIBILITY

Data are available from the iBDP Biodiversity Data Portal, the institutional repository of the German Centre for Integrative Biodiversity Research (iDiv) Halle‐Jena‐Leipzig, Germany. The data can be accessed through https://idata.idiv.de/DDM/Data/ShowData/249.

## CONFLICT OF INTEREST

None declared.
