# Supplementary material for: Different facets of tree sapling diversity influence browsing intensity by deer dependent on spatial scale
Source: Ecol Evol. 2017 Jul 26;7(17):6779–89. doi: 10.1002/ece3.3217 (PMC5587474; doi:10.1002/ece3.3217)

Supporting Information

For the article

Different facets of tree sapling diversity influence browsing intensity by deer dependent on spatial scale.

Published in *Ecology and Evolution* by

Authors: Bettina Ohse, Carolin Seele, Frédéric Holzwarth, Christian Wirth

The following Supporting Information is available for this article:

Table S1 Spearman's rank correlation among variables.

Appendix S1 Species composition – Principal coordinate analysis.

Appendix S2 Differences in forage quality among the three dominant tree species based on functional traits of leaves and buds (soluble sugars, phenolic compounds, tissue toughness, nitrogen content).

Appendix S3 Effect of environmental variables on regional-scale browsing probabilities.

Appendix S4 Community weighted mean palatability scores.

Appendix S5 Net effect of species richness on browsing proportions across two scales.

Appendix S6 Total proportion of saplings browsed per plot.

Appendix S7 Variation of relative abundances of palatable and unpalatable plant species along the species richness gradient.

Table S1 Spearman's rank correlation among variables

Table S1 Spearman's rank correlation among variables. Abundances of the three dominant species beech (*Fagus sylvatica*), ash (*Fraxinus excelsior*), and sycamore maple (*Acer pseudoplatanus*) are relative abundances.

	NSapPlot	SpecRich	Shannon	AbBeech	AbAsh	AbSyca	Long	Lat	CrownCl	Slope	Northness	Eastness	Elevation	Dist2Trail
No. of saplings per plot	1	0.68	0.54	0.08	0.20	0.32	0.07	0.13	-0.04	-0.04	0.02	0.02	-0.05	-0.09
Species richness	0.68	1	0.94	-0.08	0.27	0.50	0.21	0.20	-0.03	-0.19	0.17	0.09	-0.07	-0.02
Shannon index	0.54	0.94	1	-0.10	0.26	0.49	0.18	0.19	-0.03	-0.18	0.16	0.08	-0.05	-0.01
Abundance of beech	0.08	-0.08	-0.10	1	-0.45	-0.21	-0.19	0.05	-0.14	0.25	-0.13	-0.08	0.05	-0.15
Abundance of ash	0.20	0.27	0.26	-0.45	1	-0.11	0.05	0.01	0.00	-0.12	0.10	-0.02	0.07	0.06
Abundance of sycamore	0.32	0.50	0.49	-0.21	-0.11	1	0.19	0.16	-0.07	-0.11	0.13	0.13	0.01	-0.04
Longitude	0.07	0.21	0.18	-0.19	0.05	0.19	1	0.18	0.12	-0.38	0.35	0.29	-0.35	0.05
Latitude	0.13	0.20	0.19	0.05	0.01	0.16	0.18	1	-0.02	-0.38	0.32	0.29	-0.44	-0.08
Crown closure	-0.04	-0.03	-0.03	-0.14	0.00	-0.07	0.12	-0.02	1	-0.12	0.06	0.02	-0.06	0.13
Slope	-0.04	-0.19	-0.18	0.25	-0.12	-0.11	-0.38	-0.38	-0.12	1	-0.40	-0.23	0.07	0.05
Northness	0.02	0.17	0.16	-0.13	0.10	0.13	0.35	0.32	0.06	-0.40	1	-0.02	-0.16	0.07
Eastness	0.02	0.09	0.08	-0.08	-0.02	0.13	0.29	0.29	0.02	-0.23	-0.02	1	-0.14	0.03
Elevation	-0.05	-0.07	-0.05	0.05	0.07	0.01	-0.35	-0.44	-0.06	0.07	-0.16	-0.14	1	0.07
Distance to trail	-0.09	-0.02	-0.01	-0.15	0.06	-0.04	0.05	-0.08	0.13	0.05	0.07	0.03	0.07	1

Appendix S1 Species composition – Principal coordinate analysis

In order to capture species composition more comprehensively and consider all species' abundances, we performed a principle coordinate analysis to reduce "composition" to one or two main axes. We calculated a Bray-Curtis distance matrix using absolute abundances of all 11 species and performed a PCoA (both in R package 'labdsv'). We found that neither the three dominant species *Fagus sylvatica*, *Acer pseudoplatanus*, and *Fraxinus excelsior* nor any of the other species was clearly represented by the PCoA axes (see Figure S1 and Table S2 below). The first three axes explained only 19%, 13%, and 11.5%, respectively, of the variation in species composition. At least four axes would be needed to explain at least 50% of the variation. We concluded that this approach is not adequate to reduce species composition to two main axes for including them as proxies for species composition into the model.

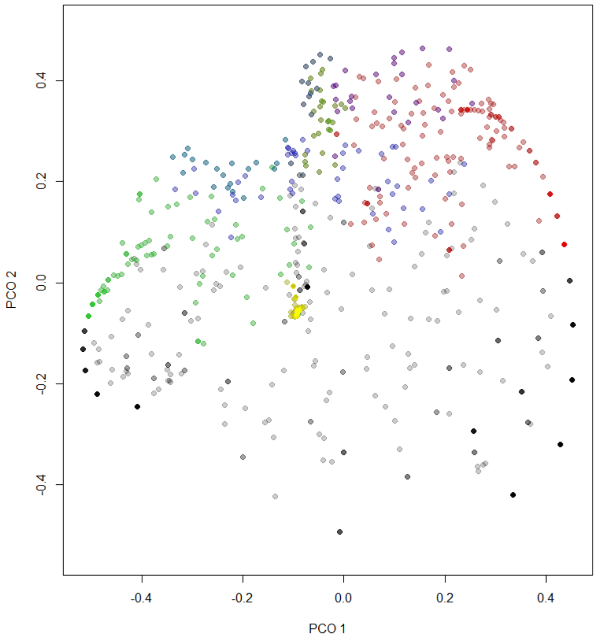

Figure S1. Absolute abundances of the 11 species across the 817 study plots.
Red – plots with > 5 *Fagus sylvatica* saplings,
blue – plots with > 5 *Acer pseudoplatanus* saplings,
green – plots with > 5 *Fraxinus excelsior* saplings,
yellow – plots with none of the three species present,
grey/black – plots with any other species composition (i.e. one or more of the three dominant species present, but with <= 5 saplings).

Table S2. Loadings of species abundances to the first three axes of the PCoA.

	Axis 1	Axis 2	Axis 3
Acer campestre	-0.096	0.039	0.058
Acer platanoides	-0.070	0.176	0.082
Acer pseudoplatanus	-0.051	0.236	0.126
Carpinus betulus	-0.078	0.135	0.040
Fagus sylvatica	0.149	0.241	-0.113
Fraxinus excelsior	-0.218	0.121	-0.078
Populus tremula	-0.126	-0.039	0.121
Prunus avium	-0.178	0.017	0.031
Querus spec.	-0.126	-0.016	0.107
Tilia spec.	0.027	0.160	0.016
Ulmus spec.	-0.118	0.107	0.062

Appendix S2 Differences in forage quality between the three dominant tree species

Table S3. Differences in browsing relevant traits of leaves and buds of the three dominant tree species in the study. Given are means (+/- standard deviation); significant differences (multiple comparison Kruskal–Wallis test, p < 0.05) between species are indicated by different letters.

Trait	*Acer pseudoplatanus*			*Fagus sylvatica*			*Fraxinus excelsior*		
Leaves									
Sugars	1.69	(+/- 0.69)	a	5.33	(+/- 1.76)	b	1.02	(+/- 0.31)	c
Nitrogen	1.87	(+/- 0.38)	a	2.11	(+/- 0.22)	ab	2.54	(+/- 0.41)	b
Phenolics									
Phenolic acids	2.11	(+/- 0.94)							
HT	50.51	(+/- 22.37)							
CT				10.05	(+/- 2.71)				
Flavonols	8.23	(+/- 3.39)	a	2.79	(+/- 1.64)	a			
Toughness	0.98	(+/- 0.14)	a	1.55	(+/- 0.31)	b	1.48	(+/- 0.67)	b
Buds									
Sugars	17.96	(+/- 6.24)	a	38.13	(+/- 12.38)	b			
Nitrogen	1.63	(+/- 0.13)	a	1.20	(+/- 0.08)	b	1.60	(+/- 0.22)	a
Phenolics									
Phenolic acids	8.22	(+/- 2.4)	a	4.47	(+/- 2.18)	a			
HT	21.51	(+/- 11.13)							
CT				9.74	(+/- 3.1)				
Bud scales	71.35	(+/- 5.40)	ab	40.26	(+/- 3.03)	a	84.76	(+/- 2.87)	b
Toughness	7.74	(+/- 1.13)	a	3.50	(+/- 1.09)	a	14.13	(+/- 2.45)	b

Sugars – total soluble sugars [mg/g fresh mass]
Phenolics – phenolic compound groups [µg/mg fresh mass], HT – hydrolysable tannins, CT – condensed tannins
Toughness – leaf and bud scale toughness, respectively [N]
Bud scales – bud scale fresh mass in relation to whole bud fresh mass [%]
Nitrogen – percent nitrogen [% dry mass]

Soluble sugars and phenolic compounds were analysed as described in (Ohse *et al.* 2016). Leaf and bud scale toughness (force to fracture) was analyzed with a punch-and-die test using a point penetrometer (Aranwela, Sanson & Read 1999) with punch diameter 1.36 mm, hole diameter 2 mm, i.e. clearance of 0.32 mm, and constant speed of 200 mm/min. We recorded the peak force needed to punch a hole through the leaf (measured in Newton, electric test stand and force gauge by SAUTER GmbH). Two measurements per individual sapling were taken. After the measurements, samples were dried at 60°C for at least 3 days (72h). For nitrogen determination, 1.5mg +/- 0.1mg of plant material was weighed into tin capsules, and analysed with a Flash2000 Elemental Analyzer (ConfloIV and Delta V advantage Isotope Ratio Mass Spectrometer, Thermo Fisher Scientific GmbH, Germany).

Differences between trait means of the three species were analysed using a Kruskal–Wallis test. This nonparametric test allows the comparison of differences among group means similar to ANOVA, but without assuming a normal distribution. We used the function 'kruskalmc' in the package 'pgirmess'

of the statistic software R, version 3.1.0 (R Core Team 2014), which accounts for multiple comparison among the three species (Siegel & Castellan 1988).

The three species differed significantly in all of the analysed traits and can thus be considered different with respect to their quality as a forage for roe deer.

References

Aranwela, N., Sanson, G. & Read, J. (1999) Methods of assessing leaf-fracture properties. *New Phytologist*, **144**, 369–383.

Ohse, B., Hammerbacher, A., Seele, C., Meldau, S., Reichelt, M., Ortmann, S. & Wirth, C. (2016) Salivary cues: simulated roe deer browsing induces systemic changes in phytohormones and defence chemistry in wild-grown maple and beech saplings. *Functional Ecology*, doi: 10.1111/1365-2435.12717.

R Core Team. (2014) R: A language and environment for statistical computing.

Siegel, S. & Castellan, N. (1988) *Non-Parametric Statistics for the Behavioral Sciences*. MacGraw Hill Int., New York, USA.

Appendix S3 Effect of environmental variables on browsing

The three environmental variables elevation, crown closure and distance to trail significantly influenced the browsing probability of plots at the regional scale (see also Table 3). Plots at relatively high elevations, under open crowns, and close to hiking trails were more likely to be browsed (Figure S2).

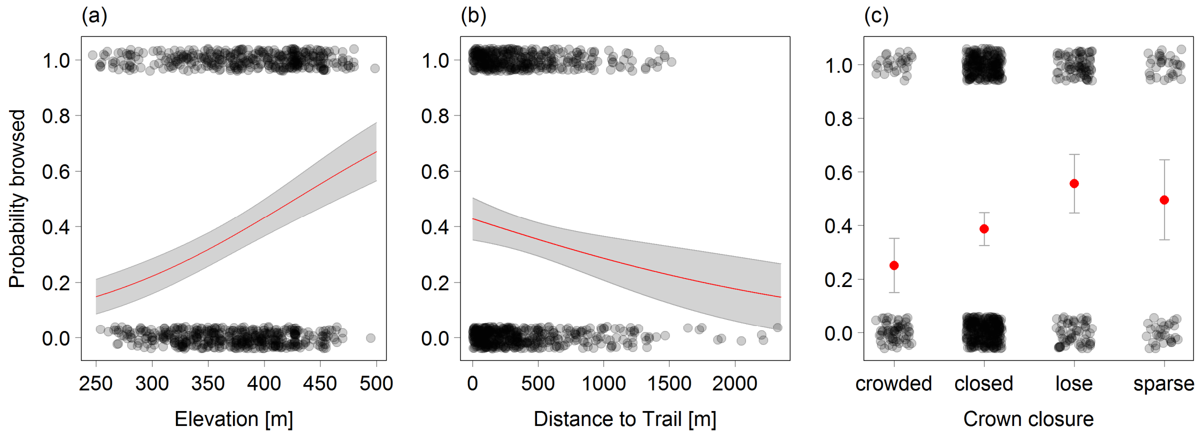

Figure S2 Regional-scale browsing probability of a plot, depending on a) elevation, b) closest distance to the next hiking trail, and c) crown closure. Dots (jittered) show the original data; red lines and dots, respectively, show predictions with 95% confidence intervals (keeping all other variables constant at their medians).

Appendix S4 Palatability scores

Based on the predicted values of browsing proportions for each species derived from the patch-scale model, we calculated the community weighted mean (CWM) palatability per plot (i.e. the sum of the predicted values of browsing proportions of all species occurring in the specific plot, weighted by the species relative abundances).

We tested whether the species composition effect at the regional scale was due to differences in overall forage quality of the plots. In the regional-scale model, we replaced species composition (relative abundances of beech and ash) by CWM palatability of each plot. The model results (Table S4) were very similar to the regional-scale final model (**Fehler! Verweisquelle konnte nicht gefunden werden.**). The variable importance ranking was almost the same, with CWM palatability replacing relative abundance of beech as the most important variable. We argue, that based on this model comparison, the relative abundance of beech was an adequate predictor for species composition and thus forage quality of a patch.

Table S4. Relative importance and summary of the coefficients for the final regional-scale model including palatability scores. Relative importance of predictors was quantified by delta AIC (change in AIC upon single term deletion, compared to the final model with AIC = 888.6). Effect sizes are standardized (continuous variables were scaled between 0 and 1). Diversity facets are in bold. CWM palatability is the community weighted mean of the species specific browsing proportions as predicted by the patch-scale model (predicted values in Fehler! Verweisquelle konnte nicht gefunden werden.**).**

Variable	delta AIC	Estimate	Std. Error	p-value
(Intercept)		-4.12	0.42	<0.001
**CWM palatability**	97.1	3.53	0.38	<0.001
Elevation	37.0	2.51	0.42	<0.001
No. of saplings per plot	28.1	6.20	1.24	<0.001
**Species richness**	16.6	2.67	0.63	<0.001
Crown closure	11.5			
closed		0.66	0.28	0.018
lose		1.32	0.33	<0.001
sparse		0.92	0.39	0.019
Distance to Trail	10.3	-1.90	0.55	<0.001

Appendix S5 Net effect of species richness on browsing proportions across two scales

The net effect of species richness on browsing proportions across the two scales was calculated as a product of the predicted values from the regional-scale model and the predicted values of the patch-scale model, keeping all other variables constant at the medians of the patch-scale. We did not calculate a net effect for *Populus tremula*, *Ulmus* spec. and *Quercus* spec. due to small sample size and thus failure of model convergence.

Considering both scales, we found that the proportion of saplings browsed decreased from monocultures to species-rich plots (Figure S3). This trend was significant (as assessed using 95% confidence intervals) for *Fagus sylvatica*, *Acer pseudoplatanus* and *Fraxinus excelsior*, and very close to significant for *Acer platanoides*, *Tilia* spec., *Carpinus betulus*, *Acer campestre* and *Prunus avium*.

Hence, although species richness had contrasting effects on browsing intensity across the two spatial scales, the net effect across both scales followed the patch-scale trend, i.e. the proportion of saplings browsed was still lower in species-rich plots.

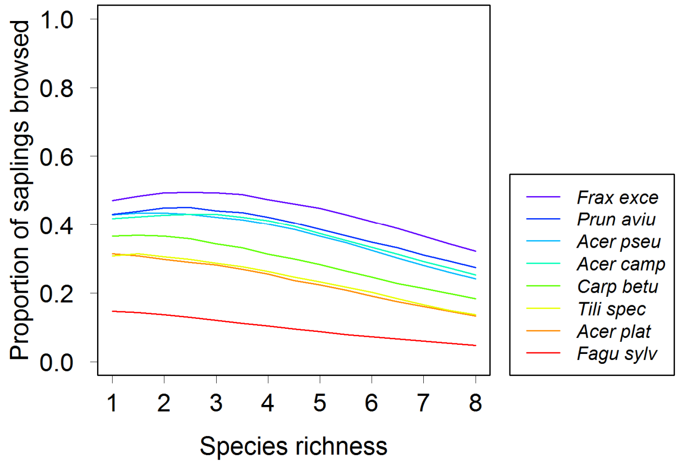

Figure S3 Net effect of species richness on the proportion of saplings browsed per species across regional and patch scale.

Appendix S6 Total browsing intensity per plot

Additionally to testing which variables influence the browsing proportions of single species within the plots, we also tested which variables influence the total proportion of all saplings browsed within a plot.

We found that, similar to the regional-scale model, species composition was the most important predictor, followed by elevation. However, similar to the results at the patch scale, species richness decreased total browsing proportions, as did high sapling numbers.

Table S5. Relative importance of predictors and summary of the coefficients for the final model predicting the total proportion of saplings browsed per plot. Relative importance of predictors was quantified by delta AIC (change in AIC upon single term deletion, compared to the final model with AIC = 1650.1). Effect sizes are standardized (continuous variables were scaled between 0 and 1). Diversity facets are in bold.

Variable	delta AIC	Estimate	Std. Error	p-value
(Intercept)		-0.218	0.214	0.301
**Species composition** (rel. abundance of beech)	32.1	-1.234	0.213	<0.001
Elevation	8.6	0.862	0.260	0.001
**Species richness**	8.2	-1.213	0.371	0.001
No. of saplings per plot	6.3	-1.389	0.464	0.003
**Species composition** (rel. abundance of ash)	4.1	0.498	0.201	0.014

Appendix S7 Variation of relative abundances of palatable and unpalatable plant species along the species richness gradient

Based on the community weighted mean palatability (see also Appendix S4), we tested whether mean plot palatability increases or decreases with species richness. We found that the community weighted mean palatability increased with species richness. This was due to both an increase in the relative abundance of the palatable species sycamore maple (*Acer pseudoplatanus*) as well as a decrease in the relative abundance of the unpalatable species beech (*Fagus sylvatica*) (Figure S4).

Figure S4 Change in palatability across the species richness gradient, depicted as the community weighted mean palatability scores as well as the relative abundance of the most dominant palatable and unpalatable species (sycamore maple, *Acer pseudoplatanus*, and beech, *Fagus sylvatica*, respectively).

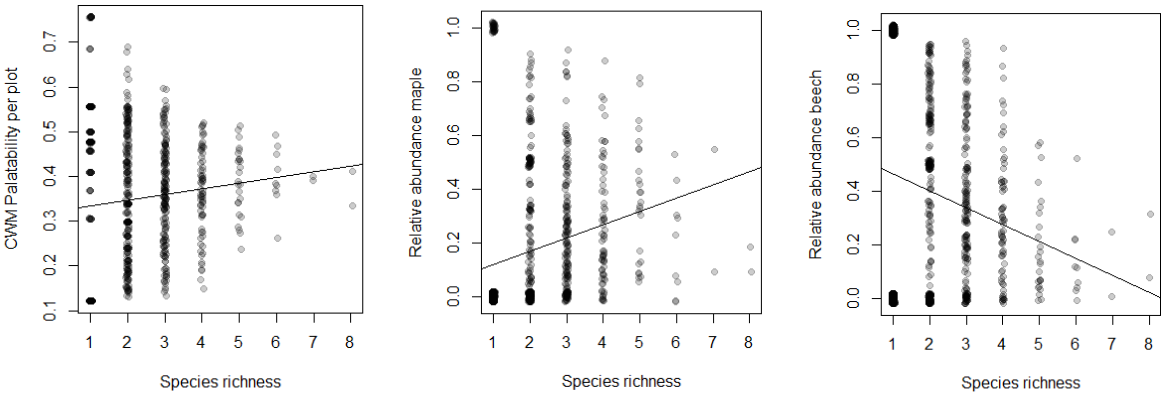

Supplement: Supplementary file 1 [file ECE3-7-6779-s001.pdf]
